# Biodiversity and structure of spider communities along a metal pollution gradient

**DOI:** 10.1007/s10646-012-0906-3

**Published:** 2012-04-28

**Authors:** Sebatian Żmudzki, Ryszard Laskowski

**Affiliations:** Institute of Environmental Sciences, Jagiellonian University, ul. Gronostajowa 7, 30-387 Kraków, Poland

**Keywords:** Metals pollution, Biodiversity, Epigeic spiders, Soil pollution, Intermediate disturbance hypothesis, Domination patterns

## Abstract

The objective of the study was to determine whether long-term metal pollution affects communities of epigeal spiders (*Aranea*), studied at three taxonomic levels: species, genera, and families. Biodiversity was defined by three indices: the Hierarchical Richness Index (HRI), Margalef index (D_M_) and Pielou evenness index (J). In different ways the indices describe taxa richness and the distribution of individuals among taxa. The dominance pattern of the communities was described with four measures: number of dominant species at a site, percentage of dominant species at a site, average dominant species abundance at a site, and the share of the most numerous species (*Alopecosa cuneata*) at a site. Spiders were collected along a metal pollution gradient in southern Poland, extending ca. 33 km from zinc and lead smelter to an uncontaminated area. The zinc concentration in soil was used as the pollution index.The study revealed a significant effect of metal pollution on spider biodiversity as described by HRI for species (*p* = 0.039), genera (*p* = 0.0041) and families (*p* = 0.0147), and by D_M_ for genera (*p* = 0.0259) and families (*p* = 0.0028). HRI correlated negatively with pollution level, while D_M_ correlated positively. This means that although broadly described HRI diversity decreased with increasing pollution level, species richness increased with increasing contamination. Mesophilic meadows were generally richer. Pielou (J) did not show any significant correlations. There were a few evidences for the intermediate disturbance hypothesis: certain indices reached their highest values at moderate pollution levels rather than at the cleanest or most polluted sites.

## Introduction

Intensive mining, metallurgy, and in previous decades the use of leaded petrol, have made contamination by a range of trace metals a common phenomenon of many industrial and urban areas. Some of these metals are not used by most organisms for any purpose and are toxic at even relatively small concentrations (e.g., Pb, As). Other play vital physiological roles (e.g., Zn, Cu, Fe, Mn) but can also be harmful when they exceed optimal concentrations. Trace metals, like other stress factors, may thus affect the natural environment, starting from effects at the level of single individuals and potentially extending to whole ecosystems. In terrestrial ecosystems, soils affected by metals have lower organic matter decomposition rates (Niklińska et al. 1998; Tyler 1976), which can lead to immobilization and accumulation of vital microelements (Giller et al. 1998), a large portion of which becomes unavailable to the first level of trophic chains–plants. This can drastically alter the productivity of plants and ecosystems (Gallagher et al. 2008) and the availability of microelements for higher trophic levels. In animals, metals affect their metabolism, physiology and life histories, and as a result may seriously compromise their fitness (Fountain and Hopkin 2004a; Kramarz and Laskowski 1997; Łagisz et al. 2002; Możdżer et al. 2003; Posthuma and van Stralen 1993; Sibly and Calow 1989). Changes in the fitness of particular species influence basic interspecies relationships (competition, predation, parasitism), and this may have serious implications for higher levels of organization-populations, communities and ecosystems (Clements and Newman 2002). Indeed, Salminen et al. (2001) showed that metal pollution can destabilize the functioning of whole ecosystems through its impact on keystone species or keystone groups: zinc contamination prevented the keystone enchytreid *Cognettia sphagnetorum* from reaching high population density and consequently led to lower microbial-mediated soil decomposition rates and soil productivity.

The epigeal spiders studied here are among the most abundant representatives of the predators’ guild on forest and meadow floors (Marc and Canard 1997) and in other environments (Duelli and Obrist 1998). They represent the second trophic level of consumers in food webs and play a crucial role in herbivore and detritivore food webs (Foelix 1996; Jung et al. 2008a). Spiders are responsible for regulation of herbivore populations, including many pests, and their absence or decrease in numbers may disturb the trophic structure and lead to excessive herbivory (Haughton et al. 1999; Kajak et al. 1968; Marc et al. 1999). For example, pest abundance was significantly lower in corn fields treated with push–pull habitat management applying higher spider richness and abundance. This novel treatment employing arthropods can serve as an alternative to synthetic insecticides (Midega et al. 2008). On the other hand, spiders are an important food source for bigger animals like birds, small mammals, amphibians, and small reptiles (Foelix 1996; Marc et al. 1999).

Spider communities—their structure, abundance and richness—are useful and recommended indicators of whole-biocenosis biodiversity (Willet 2001). Their richness correlates with the abundance of other animals (gastropods, orthopterans, carabids, birds) and can be applied in conservation ecology and assessment of environmental changes (Duelli and Obrist 1998; Sauberer et al. 2004). At the same time, spider richness and abundance strongly depend on the condition and structure of the flora (e.g., Oxbrough et al. 2007; Sauberer et al. 2004), landscape structure and landscape disturbances (Schmidt et al. 2008; Willet 2001). The vegetation—its type, height and composition—plays a vital role in ecosystems, influencing their microclimate and determining the herbivorous species community, the main source of spiders’ prey. The variability of prey, with their different life strategies, influences the diversity of spiders, which are not a strictly homogenous group of predators. There are two basic hunting strategies: “sit and wait”, and active hunting (and there are many variants). Spider species have different styles of hunting, and they hunt for different kinds of prey (Marc and Canard 1997, Marc et al. 1999). Thanks to this, their diversity can reflect the diversity of other trophic levels, indicating the spiders’ victims and the victims’ food source. Yet another advantage of using this group of predators as a diversity indicator is that they are easy to collect and the sampling techniques are simple and cheap (Jung et al. 2008a; Kapoor 2008).

Spiders also make good indicators for ecotoxicological studies. They have been shown to accumulate the highest concentrations of many trace metals among all terrestrial invertebrates (Hendrickx et al. 2004; Laskowski and Kammenga 2000). When intoxicated with metals they exhibit strong physiological reactions to the intoxication, for example by having elevated levels of detoxifying enzymes (Babczynska et al. 2006; Wilczek et al. 2003, 2004), which may lead to shifts in their energy budgets. Despite these potential advantages as indicators of contamination, the biodiversity of this group has not been assayed very often in polluted areas, and there are not many papers addressing this problem. Gargasz (2007) found a strong negative impact of elevated metal concentrations on spider abundance and biodiversity in the vicinity of a copper smelter in Poland and a nickel smelter in the United Kingdom. On the other hand, Jung et al. (2008a) demonstrated rather small differences in spider diversity and community composition in response to cadmium and lead pollution in a moderately polluted area in Korea. In other animals the biodiversity patterns in contaminated areas are not consistent either. For example, Creamer at al. (2008) showed that the abundance of earthworms (*Lumbricidae*) was reduced in response to copper contamination, *Enchytreidae* and *Nematoda* appeared sensitive to both copper and zinc, and *Collembola* only to zinc. Tischer (2005) reported decreased earthworm biodiversity and biomass in copper-contaminated areas, and Lukkari et al. (2003) found similar effects in response to pollution with a mixture of metals. In the area covered here, Skalski et al. (2010) showed earlier that the abundance and diversity of ground beetles (*Carabidae*) decreased with increasing metal pollution. In contrast, no such trend was found for oribatid mites (Skubala and Kafel 2004).

In this study we investigated the impact of long-term metal pollution on communities of epigeal spiders. We used standard biodiversity indices to define richness patterns and describe dominance structure.

## Materials and methods

### Sites, fieldwork and laboratory work

The study area is located in the Olkusz region (S. Poland), which is naturally rich in zinc and lead ores; mining and smelting activity there dates back to medieval times. The whole region has elevated soil concentrations of a number of metals, especially Zn, Pb, and Cd. The really serious pollution occurred in the 1970s when Poland’s largest Polish zinc-and-lead smelter, Bolesław, constructed in the 1960s, reached peak production and peak emissions. Although robust measures were undertaken to control emissions in 1980 and later, a large area in close proximity to the smelter and mines remains highly polluted with metals. The highest recently reported surface soil concentrations (mg kg^−1^ dry mass) were 6151 Zn, 71.4 Cd, 2206 Pb, 54.5 Cu and 18.6 Ni in forests, and 2229 Zn, 20.3 Cd, 814 Pb, 49.3 Cu and 79.5 Ni in meadows (Stefanowicz et al. 2008). Zinc levels are elevated as far as 15–20 km from the smelter (Table 1).

**Table 1:** Characteristics of research sites, and abundance of collected mature and juvenile spiders

Site code	Distance from smelter (km)	Zn concentration in soil (mg kg^−1^)	Coded Zn concentration	Mature spider abundance	Juvenile spider abundance	Juvenile proportion (%)
MX1	1.9	1350	1.396	459	106	23.09
MX2	3.7	390	0.856	1027	107	10.42
MX3	4.0	390	0.856	417	52	12.47
MX4	8.6	259	0.678	1653	83	5.02
MX6	32.9	85.5	0.197	942	64	6.79
MX5	20.0	54.3	0.000	1242	112	9.02
MM2	0.6	2229	1.416	1390	122	8.76
MM4	8.6	273	0.504	965	89	9.33
MM3	4.0	239	0.446	1435	104	7.25
MM5	20.0	145	0.229	1922	231	12.02
MM6	32.9	85.5	0.000	2683	61	2.25

Coded Zn concentration = log-transformed concentration minus the lowest value from MX or MM transect (see Methods for details)

*MX* xerothermic meadow, *MM* mesophilic meadow

For this study we demarcated two meadow transects along the pollution gradient. Since the organisms of different habitats might respond to metal pollution differently, we established one transect on xerothermic meadow (6 sites, MX1 to MX6) and another on mesophilic meadow (5 sites, MM2 to MM6). These meadow types are representative for this region and differ in abiotic (humidity, temperature, soil permeability) and biotic (vegetation type) conditions. Xerothermic meadows are considered harsher environments: they are drier and warmer, with larger daily temperature amplitudes, and are less fertile than mesophilic meadows, which are usually described as more benign.

Metal concentrations in the soil at all study sites were determined earlier by Stefanowicz et al. (2008). The Zn concentrations are reported in Table 1. The reference site for xerothermic meadow is ca. 20 km from the pollution source, and the one for mesophilic meadow ca. 33 km from it (Table 1).

At each site a rectangular sampling plot measuring 5 × 10 m was established, and 66 Barber pitfall traps were placed 1 m apart in the plot. The traps were 150 ml plastic vendor cups half-filled with preserving liquid (ethane-1,2-diol, Petrygo and Borygo automotive antifreeze, Poland). Material from all traps at each site was pooled in one container to make one sample.

Sampling was done six times in 2006: 10, 21 and 27 May, and 3, 11 and 27 June. Sampling was done in May and June because spring and early summer is the time of highest activity of terrestrial spiders. It is the mating season. The males are roaming and seeking females, and the females are looking for suitable hideouts where they can stay later with cocoons. At site MX3 no sample was obtained on May 27 because the pitfall traps had been destroyed, so 65 samples were collected in total. Material collected from each site during the whole sampling period was considered one sample. All collected samples were put into containers with ethanol and refrigerated for taxonomic identification. In the laboratory the spiders were separated from other material and counted. Mature individuals were identified to species level using available sources (Nentwig et al. 2011; Nieuwenhuys 2011; Platnick 2011; Roberts 1995). Because it is hardly possible to identify species in juveniles, all juveniles were considered one group, and in further biodiversity analysis only mature spiders were used. Such a procedure does not change the final results significantly (Sackett et al. 2008).

### Biodiversity measures and dominance structure

On the basis of species identification, for each taxonomic level (species, genus, family) three biodiversity indices were calculated: Hierarchical Richness Index (HRI), Margalef index (D_M_) and Pielou evenness index (J). The Pielou index measures how evenly individuals are distributed among taxa in a community, D_M_ reports the number of species corrected for sample abundance, and HRI summarizes both aspects of community structure in one comprehensive measure and is recommended specifically for ecotoxicological research (French and Lindley 2000). It takes into account the number of species, their abundance, and the distribution of individuals among taxa. All indices were calculated with Excel spreadsheet, and are defined with the following formulae:

$$ HRI = \sum\limits_{i = 1}^{S} {(i \times n_{i} )} $$

$$ D_{M} = \frac{S - 1}{\ln N} $$

$$ J = \frac{{ - \sum\nolimits_{i = 1}^{S} {p_{i} \ln p_{i} } }}{\ln S} $$

where *i* is the rank of a taxon (according to its abundance in a sample; most abundant—rank 1), *n*_*i*_ is the abundance of taxon *i*, *S* is the number of taxa in a sample, *N* is the total number of specimens, and *p*_*i*_ is the proportion of taxon *i* in a sample.

Dominance structure was analyzed using Czachorowski’s classification (Czachorowski 2006). Only Czachorowski’s two main classes were taken into account: eudominants (comprising more than 10 % of a sample) and dominants (comprising from 5.1–10 % of a sample). For statistical analysis the two classes were combined into one group of dominants. The following dominance indices were calculated: number of dominant species at a site (DSN), percentage of dominant species at a site (DSSP = 100 × dominant species abundance/sample abundance), and average dominant species abundance at a site (ADSA = DSSP/DSN). We also calculated the share of the most abundant species, *Alopecosa cuneata*, in samples (AcP = *Alopecosa cuneata* abundance in sample/total sample abundance).

### Statistics

The relationships between pollution level and diversity and dominance indices were tested with regression analysis, with the soil concentration of zinc (mg Zn kg^−1^ dry mass, hereinafter referred to as “soil Zn”) as the independent variable. Zinc was chosen as a sole pollution index because in the previous work in the same area the concentrations of all major metal pollutants in the soil were found to be highly correlated (Stefanowicz et al. 2008). The two transects, xerothermic and mesophilic, were analyzed together, with comparison of intercepts and slopes: a significant difference in intercepts would mean differences in spider biodiversity between the two meadow types with no pollution, while differences in slopes would mean differences in vulnerability to pollution between the spider communities of the two meadow types. The data on Zn concentrations were taken from Stefanowicz et al. (2008, Table 1) and were log_10_-transformed. Comparison of intercepts requires both regressions to start from zero on the log-transformed axis, so from each log-transformed concentration we subtracted the zinc concentration level of the control site for that transect. For each transect we assessed the relationships between soil Zn and the following characteristics of spider communities: (i) total number of spiders; (ii) proportion of juveniles; (iii) biodiversity of mature spiders (HRI, D_M_, J for each of the three taxonomic levels); (iv) dominance indices (DSN, DSSP, ADSA for each of the three taxonomic levels); and (v) *Alopecosa cuneata* abundance in the sample vs. total sample abundance (AcP). Then we compared the regression lines between the meadow types by the dummy variable method. This procedure is based on constructing indicator (dummy) variables which allow testing of whether a single model can be used across groups. With only two groups (meadow types) compared, the linear regression model is:

$$ Y = a_{1} + b_{1} X + Ia_{2} + Ib_{2} X $$

where the indicator variable *I* takes the value 0 if false (meadow type 1) and 1 if true (meadow type 2). In effect, this procedure fits a separate line for each level of the categorical variable and allows comparison of intercepts and slopes between the levels of that variable. Significance of *a*_*2*_ indicates significant differences in intercepts, while significance of *b*_*2*_ means significantly different slopes.

All statistical analyses were done with Statgraphics Centurion XV software (Manugistics Company, Rockville, MD, U.S.A.).

## Results

### Number of specimens and fraction of juveniles. Species, genus and family richness

In total, 1131 juvenile and 14,135 mature spiders were collected (Table 1). Spiders were more numerous in mesophilic (9036 mature, 608 juveniles; Table 1) than in xerothermic meadows (6264 mature, 524 juveniles; Table 1). There was no clear trend in spider numbers along the pollution gradient. None of the linear regressions for either meadow type were significant (*p* = 0.12 for mature spiders, *p* = 0.13 for juveniles). In xerothermic meadows, spiders belonged to 70 species, 36 genera and 16 families, and in mesophilic meadows to 61 species, 32 genera and 14 families (Table 2). There was no clear trend for these values, and no statistical analysis was performed because the number of taxa is better reflected by the Margalef index used in further analyses.

**Table 2:** Community structure at research sites

Site	Number of				DSSP (%)	ADSA (%)	*Ac*P (%)
	S	G	F	DSN			
MX1	46	28	15	8	60.58	7.57	14.38
MX2	40	25	13	3	71.57	23.86	31.35
MX3	40	28	13	4	58.75	14.69	29.98
MX4	38	23	12	4	73.98	18.50	52.69
MX6	39	23	12	4	62.72	15.68	22.40
MX5	47	24	12	4	59.56	14.89	28.90
MM2	42	26	15	4	76.88	19.22	13.07
MM4	44	23	14	5	72.14	14.43	10.47
MM3	45	24	14	7	70.10	10.01	8.36
MM5	46	26	12	4	63.95	15.99	31.22
MM6	32	18	11	6	77.09	12.85	14.13

*S* number of species, *G* genera, *F* families, *DSSP* percentage of dominant species at site, *DSN* number of dominant species at site, average dominant species abundance at site (ADSA = DSSP/DSN), share of the most abundant species at site (AcP)

Sites are ordered from least (low numbers) to most polluted (high numbers) for each transect (*MX* xerothermic meadow, *MM* mesophilic meadow)

### HRI, Margalef and Pielou indices

**Fig. 1:**
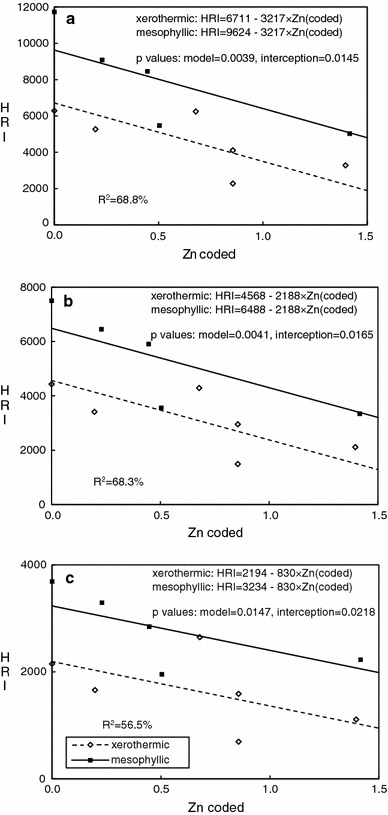
Relationship between soil Zn and HRI for species (**a**), genera (**b**) and families (**c**) for two types of meadows. For Zn concentration coding see Methods and Table 1. Slopes were assumed to be equal, as the *p* values for differences between slopes were 0.36 for species, 0.36 for genera and 0.78 for families

The HRI values for all taxonomic levels decreased with increasing soil Zn in both meadow types (Table 3). Linear regression analyses revealed differences only in intercepts, with lower values for xerothermic habitats, and not in slopes (vulnerability). Under the assumption of equal slopes, the relationships were negative and linear between soil Zn and HRI for species (*p* = 0.039), genera (*p* = 0.0041), and families (*p* = 0.0147) (Table 2, Figure 1). The significant difference in intercepts for species (*p* = 0.0145), genera (*p* = 0.0165) and families (*p* = 0.0218) show that the two investigated meadow types differed in biodiversity level as described by HRI.

**Table 3:** Biodiversity indices at three taxonomic levels for spider communities along the metal pollution transect

	HRI			Margalef index			Pielou index		
	S	G	F	S	G	F	S	G	F
MX1	3277	2113	1111	7.34	4.41	2.28	0.81	0.77	0.6
MX2	4104	2953	1591	5.62	3.46	1.73	0.61	0.54	0.34
MX3	2273	1491	695	6.46	4.48	1.99	0.73	0.69	0.42
MX4	6247	4291	2649	4.99	2.97	1.48	0.55	0.54	0.40
MX6	5265	3415	1660	5.55	3.21	1.61	0.73	0.73	0.47
MX5	6285	4431	2151	6.46	3.23	1.54	0.68	0.68	0.45
MM2	5030	3344	2229	5.66	3.45	1.93	0.56	0.50	0.34
MM4	5470	3561	1955	6.26	3.20	1.89	0.7	0.70	0.53
MM3	8460	5909	2846	6.05	3.16	1.79	0.73	0.74	0.53
MM5	9080	6451	3295	5.95	3.31	1.45	0.64	0.64	0.44
MM6	11730	7500	3690	3.93	2.15	1.27	0.68	0.62	0.32

Sites are ordered from least (lowest numbers) to most polluted (highest numbers) for each transect (*MX* xerothermic meadow, *MM* mesophilic meadow)

*S* species, *G* genus, *F* family

**Fig. 2:**
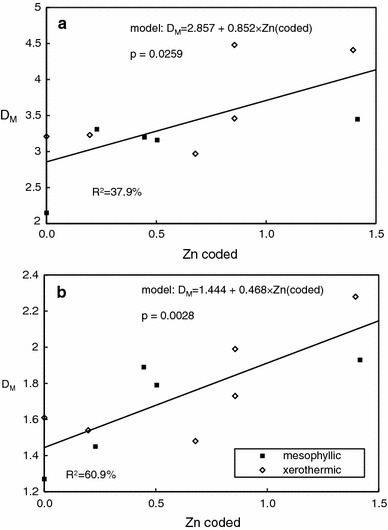
Relationship between soil concentration of soil Zn and Margalef index (D_M_) for genera (**a**) and families (**b**) for two types of meadows. For Zn concentration coding see Methods and Table 1. Slopes and intercepts were assumed to be equal, as the *p* values for differences between meadows were all >0.16

Unlike HRI, the Margalef D_M_ index increased with increasing soil contamination (Table 3). Full linear regression analyses, including slope and intercept comparisons between meadow types, revealed no significant relationship for species (*p* = 0.575) and a marginally significant one for genera (*p* = 0.0852). For the latter case, as the *p* value was <0.1 we repeated the regression analysis assuming equal slopes and intercepts, since there were no differences between the meadow types in this respect. With this assumption the analysis yielded a significant (*p* = 0.0259) positive relationship between the soil Zn and D_M_ for genera (Table 4, Figure 2). The relationship between Zn contamination and biodiversity measured at the family level was significant at *p* = 0.0471, again with no differences between slopes or intercepts. Consequently, we fit one common regression to both meadow types; this gave slightly higher significance for the relationship (*p* = 0.028; Table 4, Figure 2). The results thus indicated no differences in biodiversity or vulnerability as measured by D_M_ between the two investigated meadow types, and an increase of D_M_ with contamination for families and genera.

**Table 4:** Linear regression analysis of relationships between soil log(Zn) and community diversity indices

	Taxonomic level		F	*p*-value
HRI	Species	Model	12.03	0.0039
		Intercept difference	9.66	0.0145
		R^2^	68.8 %	
	Genus	Model	11.77	0.0041
		Intercept difference	9.14	0.0165
		R^2^	68.3 %	
	Family	Model	7.5	0.0147
		Intercept difference	8.07	0.0218
		R^2^	56.5 %	
Margalef index	Genus	Model	7.09	0.0259
		R^2^	37.9 %	
	Family	Model	16.58	0.0028
		R^2^	60.9 %	

Only regressions significant at *p* ≤ 0.05 are reported

*HRI* hierarchical richness index

There was no significant relationship between soil Zn and the Pielou (J) index (Table 3). The *p* values were high for species (0.61), genera (0.70) and families (0.90).

In the raw data we see a slight elevation of almost all indices from the moderately polluted plots (Table 3). This may indicate some hormetic effect or confirm intermediate disturbance hypothesis (IDH); we did not test its statistical significance formally but it certainly deserves to be discussed, as will be done further on.

### Dominance structure

In both types of meadows the most dominant species at all 11 sites was *Alopecosa cuneata*. Also frequent at all sites were *Alopecosa pulverulenta* and *Aulonia albimana*. *Xerolycosa miniata* and *Pardosa pullata* were noted as dominants in four instances. All these species belong to the *Lycosidae* family and are considered common and eurytopic. All were numerous in the vicinity of the smelter (Tables 2, 3).

Regression analyses did not show a significant relationship for *A. cuneata* frequency (AcP; *p* = 0.33) or for number of dominant species on site (DSN, *p* = 0.34). Nor did DSSP and ADSA show a relationship to contamination level in either type of meadow (*p* = 0.36 and *p* = 0.64, respectively; Table 2). Despite the lack of statistical significance, the raw data suggested patterns in both indices for xerothermic meadow: (i) average dominant species abundance (ADSA) decreased, (ii) number of dominant species on site (DSN) increased, and (iii) total species number (S) increased with the increase of pollution (Table 2), except for site MX2 which was more humid than the others. In mesophilic meadows all the indices fluctuated randomly, but the least contaminated site (MM6) showed the fewest species (32) and a high number of dominants (6).

## Discussion

Despite the lack of any relationship between pollution and the total abundance of spiders or the frequency of juveniles, HRI decreased significantly with increasing soil Zn for all taxonomic levels (species, genera, families). Gargasz (2007) observed the same pattern of diversity measured by HRI in his study of copper- and nickel-contaminated sites in Poland and the UK. Using the Shannon diversity index (H’) in studies of spider communities inhabiting metal-polluted sites in Korea, Jung et al. (2008a) also noted a slight, nonsignificant decrease of H’ with increasing lead and cadmium concentrations, probably because the pollution level was much lower than at the Polish sites: in Korea the highest concentrations were 45.6 mg/kg Pb and 1.39 mg/kg Cd, against 814 mg/kg Pb and 20.3 mg/kg Cd in Poland. There was no zinc contamination at the Korean sites; in our study area it was the main contaminant. In this context it is worth pointing out that in work done in the Avonmouth area, contaminated with a mixture of metals very similar to that at the Olkusz sites, Spurgeon and Hopkin (1996) concluded that zinc was the main factor responsible for limiting the distribution of earthworms in the surroundings of the smelter.

Besides pollution, the type of habitat also had an important effect on HRI biodiversity, with xerothermic meadows exhibiting lower spider diversity. This is not unexpected: in terms of humidity, temperature amplitude, insolation and flora composition, xerothermic meadows are harsher environments (Clements and Newman 2002). Such places are characterized by relatively high natural environmental stress, which limits biodiversity and steers communities towards fewer but more specialized species.

Unlike HRI, Margalef D_M_ increased with pollution level for genera and families. No trend for species *D*_M_ was found, and there were no differences between meadow types. Gautman et al. (2006) reported similar results in a study of five arthropod orders. They noted the highest *Coleoptera* species richness at polluted industrial sites, and six of the species they collected were found only at such sites. Fountain and Hopkin (2004a) confirmed the same pattern for *Collembola* communities in polluted areas. One explanation of this fairly consistent pattern might be that pollutant-caused changes in environmental conditions can lead to the emergence of new niches for new species, as described in detail in work on sulphur dioxide and *Heteroptera* (Brändle et al. 2001, 2006): higher SO_2_ deposition had altered the soil conditions, making it possible for new species of plants to appear in the area. In turn, the new plant species provided a food source for new monophagous species of *Heteroptera*, and new herbivore arthropods attracted new narrowly specialized arthropod predators. Other explanation is that more resistant new species may appear in contaminated areas and outcompete species inhabiting parts of niches. The frequency of rare species can increase, as clearly reflected in the dominance structure we uncovered. More work is needed to confirm whether newcomers and certain rare species are indeed more resistant to pollution and can compete more successfully in harsh environments. Gargasz (2007) obtained different results in copper- and nickel-polluted areas, where D_M_ for genera decreased with increasing pollution. Again, the explanation may be higher copper toxicity (Pedersen et al. 1999).

At moderately polluted sites the Pielou (J) and HRI indices increased slightly, probably due to a stimulatory, hormetic effect of low stress. In the same area a similar hormetic effect was described in oribatid mites (Skubała and Kafel 2004). Under moderate pollution, organisms may not only efficiently repair any toxicant-caused damage but also overcompensate the disturbance with elevated vitality and fitness (Calabrese and Baldwin 2002; Chapman 2002). The increased Pielou index in our study may have a simpler explanation. At the moderately polluted sites the fraction of dominants decreased; recedent species may cope more efficiently with concurrent species in such a changed setting. As a result their fraction increases, evening out the distribution of individuals among species. A particular type of hormesis has been seen under other kinds of anthropogenic stresses: biodiversity was slightly higher in environments that were moderately physically disturbed (and not polluted) than in natural conditions. These disturbances involved agriculture and landscape changes (Jung et al. 2008a) or urbanization (Alaruikka et al. 2002). The effect was originally suggested in the intermediate disturbance hypothesis (IDH), describing a phenomenon found in work on rainforest spider biodiversity (Connell 1978). Elevated biodiversity in areas with dense field boundaries fits this category of explanation (Haughton et al. 1999), though not disturbance but rather high landscape tessellation is responsible here.

In this analysis neither the abundance of adult spiders nor the fraction of juveniles were related to the pollution level. Gargasz (2007) found that adult spider abundance decreased significantly with increasing pollution level in the vicinity of copper and nickel smelters, and a number of studies of other groups of invertebrates have shown abundance decreasing with pollution level, for example in *Lumbricidae*, *Enchytreidae* and microarthropods (Lukkari et al. 2003). The discrepancy between those studies and ours may be due to differences in sampling methods, field conditions, groups of investigated animals, or type of pollution. All the taxonomic groups in those papers except for the one examining spiders (Gargasz 2007) are soil-dwelling animals, and as such are exposed to contaminants not only through food but directly through soil solution. It is known that metals basically are toxic only when they occur in dissolved ionic forms (Fountain and Hopkin 2004a), so animals exposed through soil solution are more exposed to their toxicity. Also the type of metal is important, as shown in a number of studies. Zinc and lead exhibit relatively low toxicity in invertebrates, and cadmium, although more toxic, is usually present at low concentrations even in highly polluted soils (Hopkin and Hames 1994). Gargasz (2007) made his studies in a copper mining and smelting area where the main pollutant was Cu. Although essential at low concentrations, this metal is known to be one of the most toxic elements (Pedersen et al. 1999), and compounds of it have been used for centuries as pesticides.

Although we concentrated so far on possible direct effects of pollution on spider communities, it should be stressed that also a range of secondary, indirect effects are possible.

Relationships between organisms in a community may be massively influenced without any direct effects on certain groups (Lefcort et al. 2002) and can lead to unpredictable changes at the community level (Geiszinger et al. 2009). These phenomena are of particular interest in modern ecotoxicology and ecological risk assessment (Arapis 2005), but are still poorly recognized, and available data focus mainly on aquatic ecosystems (Fleeger et al. 2003).

In food webs spiders are predators, prey and competitors. The spiders investigated herein are general epigeic predators which feed mostly on collembolans, hymenopterans and hemipterans what places them in the detritus and grazer food webs (Nentwig 1982; Foelix 1996). Both of these webs are affected by metal pollution, and it was reported in many studies that potential prey of the spiders can be directly affected by metals, what results in a decrease of their abundance (e.g., Fountain and Hopkin 2004a, Nahmani and Rossi 2003). The lack of prey can be, in turn, reflected in the decrease of predators’ numbers what would result in patterns similar to those observed in this study for spiders.

Indirect effect on competition in terms of species reordering within epigeic *Araneae* group was briefly mentioned in the first part of discussion. It can involve, however, also other competitors. The main ground-dwelling spiders’ competitors are coleopterans, especially *Staphylinidae* and *Carabidae* families. Carabids were investigated in the same research area and showed similar pattern of changes in biodiversity (Skalski et al. 2010) as epigeic spiders. This observation suggests that indirect effects via competition with carabids probably did not occur, but effects of other competitors cannot be excluded.

The present study does not allow for separating direct and indirect effects of metal pollution on epigeic spiders. However, significant effects of pollution on spider communities have been proved and further research would be required to find out exact causes of the observed patterns.

## Conclusions

1. HRI-measured diversity for species, genera and families significantly decreased with metal pollution level in both xerothermic and mesophilic meadows. Diversity differed between the two meadow types. There were no differences in the vulnerability of spider communities to contamination.
2. The Margalef index for genera and families increased with increasing contamination.
3. There was evidence for hormesis or the intermediate disturbance hypothesis.
4. No statistically significant changes in the dominance structure were found, but in xerothermic meadows there was some increase of total species number and dominant species number at heavily polluted sites.
5. Metal pollution did not affect the Pielou index.
6. Metal pollution did not affect spider abundance or the fraction of juveniles.
7. Of the indexes applied, HRI was the one most sensitive to the effects of pollution on community structure at different levels of taxonomic resolution.
8. Spiders community may be affected by heavy metals not only directly, but also by indirect effect of pollutants.
